# Mitochondrial replication's role in vertebrate mtDNA strand asymmetry

**DOI:** 10.1098/rsob.230181

**Published:** 2023-12-20

**Authors:** André Gomes-dos-Santos, Nair Vilas-Arrondo, André M. Machado, Esther Román-Marcote, Jose Luís Del Río Iglesias, Francisco Baldó, Montse Pérez, Miguel M. Fonseca, L. Filipe C. Castro, Elsa Froufe

**Affiliations:** ^1^ CIIMAR/CIMAR - Interdisciplinary Centre of Marine and Environmental Research, University of Porto, Matosinhos, Portugal; ^2^ Programa de Doctorado ‘Ciencias marinas, Tecnología y Gestión’ (Do*MAR), Universidad de Vigo, Vigo, Spain; ^3^ Centro Oceanográfico de Vigo (COV), Instituto Español de Oceanografía (IEO-CSIC), Subida a Radio Faro, 50, Vigo (Pontevedra), 36390, Spain; ^4^ Centro Oceanográfico de Cádiz (COCAD), Instituto Español de Oceanografía (IEO-CSIC), Puerto Pesquero, Muelle de Levante s/n, Cádiz, 11006, Spain; ^5^ Department of Biology, Faculty of Sciences, University of Porto, Porto, Portugal

**Keywords:** mitogenome, strand asymmetry, deep-sea fish, mitochondrial gene order, rearrangements

## Abstract

Mitogenomes are defined as compact and structurally stable over aeons. This perception results from a vertebrate-centric vision, where few types of mtDNA rearrangements are described. Here, we bring a new light to the involvement of mitochondrial replication in the strand asymmetry of the vertebrate mtDNA. Using several species of deep-sea hatchetfish (Sternoptychidae) displaying distinct mtDNA structural arrangements, we unravel the inversion of the coding direction of protein-coding genes (PCGs). This unexpected change is coupled with a strand asymmetry nucleotide composition reversal and is shown to be directly related to the strand location of the Control Region (CR). An analysis of the fourfold redundant sites of the PCGs (greater than 6000 vertebrates), revealed the rarity of this phenomenon, found in nine fish species (five deep-sea hatchetfish). Curiously, in Antarctic notothenioid fishes (Trematominae), where a single PCG inversion (the only other record in fish) is coupled with the inversion of the CR, the standard asymmetry is disrupted for the remaining PCGs but not yet reversed, suggesting a transitory state. Our results hint that a relaxation of the classic vertebrate mitochondrial structural *stasis* promotes disruption of the natural balance of asymmetry of the mtDNA. These findings support the long-lasting hypothesis that replication is the main molecular mechanism promoting the strand-specific compositional bias of this unique and indispensable molecule.

## Graphical abstract

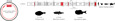

## 1. Introduction

Counterintuitively, the mitochondrial genome (mtDNA) is far more variable than normally recognized, including structure, gene content, order and orientation, organization and mode of expression [1]. In Metazoa, several types of mtDNA are described, including linear telomeric molecules (e.g. Cnidaria), ‘giant’ circular molecules (e.g. some Bivalvia and Placozoa) and several mini-circular molecules (e.g. Insecta) [1,2]. In vertebrates, the classic mtDNA is described as a compact circular molecule, maternally inherited, between 16–19 kbp and with two compositionally distinct strands, conventionally referred to as heavy (H-strand with high G composition) and light (L-strand with low G composition) [2–4]. The standard vertebrate mtDNA encodes 37 genes, 13 protein-coding genes (PCGs); two ribosomal RNAs (rRNAs); 22 transfer RNAs (tRNAs); and two differently located and strand-specific replication origins (heavy strand replication origin [oriH] and light strand replication origin [oriL]) [2–5]. The oriH is placed within a larger non-coding region, the Control Region (CR), which includes several regulators of the mtDNA replication and transcription and where four evolutionarily conserved sequence blocks (CSBs) are commonly found (i.e, CSB-I, CSB-II, CSB-III and CSB-D) [2–5]. The 13 mtDNA PCGs, which follows the standard architecture (i.e. number, relative positioning and coding strand), play key functional roles in the oxidative phosphorylation (OXPHOS) cascade [2–4].

Exceptions to this generalized picture in vertebrate classes are extremely rare, with very few and small-scale deviations [1,6–10]. Among the different groups, birds and reptiles show the highest distribution of rearranged genes, while mammals and fish show residual examples of rearrangements [1,6,8]. In fish, three distinct types of mtDNA gene rearrangements have been described recently by Satoh *et al*. [11]: ‘shuffling’, i.e. local position change maintaining coding polarity (i.e. genes coding strand); ‘translocation’, i.e. movement to a distinct location maintaining the genes encoded strand; and the rarest event ‘inversion’, i.e. genes (or non-coding unities) switch to their complementary strand [1,12–17]. Inversions, when acting upon the replication controlling unities (i.e. the CR or oriH), have been shown to promote a switch of the strand asymmetry nucleotide composition. Consequently, the mtDNA replication is suggested to be involved in the differently accumulated strand mutation patterns of the mtDNA (assuming an asymmetric model of mtDNA replication) [1,12,15].

Marine deep-sea hatchetfish from the family Sternoptychidae (Order: Stomiiformes) are a group of small (less than 100 mm) and peculiar mesopelagic fishes [18]. The family consists of two subfamilies, Maurolicinae and Sternoptychinae, which include around 70 species distributed through 10 genera [18–20]. These species are generally found in high abundance and biomass within the mesopelagic ichthyofauna and with important ecological functions and a key trophic position [21,22]. Species from the family Sternoptychidae have been described as *some of the most bizarre stomiiforms* and are characterized by having a condensed body with a reflecting flattened silver side that allows camouflage [23]. As in all other Stomiiformes, deep-sea hatchetfish possess specialized bioluminescent organs, i.e. photophores that allow them to produce light [23–25].

Here, we describe the structure of Sternoptychidae mtDNA which show a diverse and unusual gene architecture. Our findings include mtDNA gene shuffling, translocation, and inversions, acting on PCGs, tRNA rRNA and/or CR. In particular, we demonstrate that strand asymmetry nucleotide composition reversal occurs when genes change their coding polarity relative to the CR (i.e. oriH, initiation of replication). Conversely, inversions of CR were shown to promote the complete nucleotide strand asymmetry reversal in two deep-sea hatchetfish species. Moreover, by investigating over 6000 species we determine that strand asymmetry is a rare event in vertebrate mtDNAs. In Antarctic notothenioid fishes (Trematominae), an inversion in a coding region (the only case previously reported in fish) coupled with the CR inversion is shown to disrupt the standard asymmetry for the remaining PCGs. Together, our findings provide strong evidence supporting the long-lasting theory that replication is the main molecular mechanism promoting the strand-specific compositional bias of the mtDNA.

## 2. Results and discussion

### 2.1. Deep-sea hatchetfish mtDNA show a widespread complex repetitive region hampering full sequence circularization

We reconstruct four mitochondrial sequences from marine hatchetfish: *Argyropelecus aculeatus* Valenciennes, 1850 (lovely hatchetfish), *Argyropelecus hemigymnus* Cocco, 1829 (half-naked hatchetfish), *Argyropelecus olfersii* (Cuvier, 1829) and *Maurolicus muelleri* (Gmelin, 1789) (Mueller's pearlside). The raw sequencing reads and mtDNA assemblies were deposited at NCBI, and respective SRA and assembly accessions are depicted in table 1 and linked to BioProject PRJNA977192.

**Table 1.** List of whole mitogenomes used in the whole mitogenome-based phylogeny.

family	species	GenBank accession	BioSample accession	SRA accession	mtDNA length (bp)
Sternoptychidae	*Argyropelecus aculeatus*^a^	OR062951	SAMN35453525	SRR24764805 and SRR24764804	23 291
Sternoptychidae	*Argyropelecus affinis*	AP012964.1	—	—	15 489
Sternoptychidae	*Argyropelecus hemigymnus*^a^	OR062952	SAMN35453526	SRR24764802	20 747
Sternoptychidae	*Argyropelecus olfersii*^a^	OR062953 and OR062954	SAMN35453527	SRR24764803	16 414
Sternoptychidae	*Maurolicus muelleri*	AP012963.1	—	—	15 230
Sternoptychidae	*Maurolicus muelleri*^a^	OR062955	SAMN35453528	SAMN35453528	16 604
Sternoptychidae	*Polyipnus polli*	AP012962.1	—	SRR24764801	16 773
Sternoptychidae	*Sternoptyx diaphana*	MT588184.1	SAMN35453678	SRR24764797-SRR24764800	17 224
Sternoptychidae	*Sternoptyx obscura*	OP057081.1	—	—	18 293
Gonostomatidae	*Sigmops gracilis*	AB016274.1	—	—	16 436
Stomiidae	*Tactostoma macropus*	LC377784.2	—	—	17 563
Stomiidae	*Photonectes margarita*	AP018417.1	—	—	18 592
Stomiidae	*Trigonolampa miriceps*	AP012961.1	—	—	15 709
Stomiidae	*Astronesthes lucifer*	AP012959.1	—	—	15 491
Stomiidae	*Stomias atriventer*	MG321595.1	—	—	17 596
Stomiidae	*Chauliodus sloani*	AP002915.1	—	—	17 814
Diplophidae	*Diplophos taenia*	AP012960.1	—	—	16 427
Diplophidae	*Diplophos sp.*	AB034825.1	—	—	16 418
Phosichthyidae	*Vinciguerria nimbaria*	AP006769.1	—	—	16 741
Phosichthyidae	*Vinciguerria nimbaria*	AP012958.1	—	—	16 741
Myctophidae	*Myctophum affine*	AP002922.1	—	—	16 239
Trachipteridae	*Trachipterus trachipterus*	AP002925.1	—	—	16 162
Ateleopodidae	*Ateleopus japonicus*	AP002916.1	—	—	16 650
Synodontidae	*Saurida undosquamis*	AP002920.1	—	—	15 737
Salmonidae	*Coregonus lavaretus*	AB034824.1	—	—	16 737

^a^Represent mitogenomes sequenced in this study.

**Figure 1.**
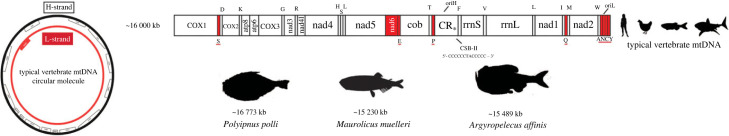
Left: schematic of circular mitochondrial molecule highlighting the protein-coding genes encoded in the different strands. Right: representation of linearized mitochondrial molecule, depicting the standard vertebrate gene order shared by the deep-sea hatchetfish species, *Polyipnus polli*, *Maurolicus muelleri* and *Argyropelecus affinis*. Genes encoded in the L-strand are depicted in red; genes encoded in the H-strand are depicted in white. *Control region has not been sequenced in either *Maurolicus muelleri* or *Argyropelecus affinis*. The CSB-II sequence is a representation of the generally expected composition of the motif.

**Figure 2.**
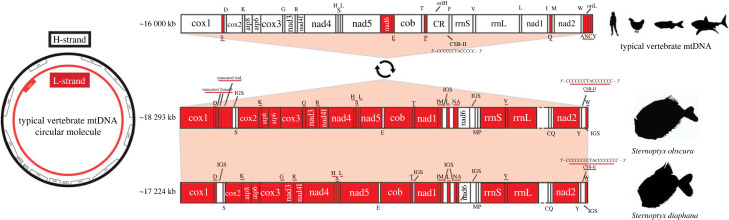
Left: schematic of circular mitochondrial molecule highlighting the protein-coding genes encoded in the different strands. Right: representation of linearized mitochondrial molecule, depicting the standard vertebrate gene order (middle) comparatively to deep-sea hatchetfish species, *Sternoptyx obscura*, *Sternoptyx diaphana*. Genes encoded in the L-strand are depicted in red; genes encoded in the H-strand are depicted in white; red shadow represents macrosyntenic patterns of gene inversion. The CSB-II sequence for the typical vertebrate mtDNA is a representation of the generally expected composition of the motif. The CSB-II sequences for the marine hatchetfish are the ones here identified.

**Figure 3.**
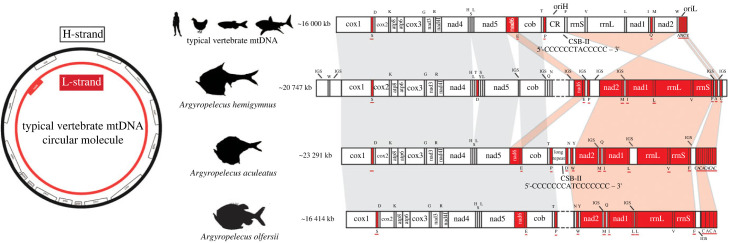
Left: schematic of circular mitochondrial molecule highlighting the protein-coding genes encoded in the different strands. Right: representation of linearized mitochondrial molecule, depicting the standard vertebrate gene order (second from the top) comparatively to deep-sea hatchetfish species, *Argyropelecus hemingymnus*, *Argyropelecus aculeatus* and *Argyropelecus olfersii*. Genes encoded in the L-strand are depicted in red; genes encoded in the H-strand are depicted in white; red shadow represents macrosyntenic patterns of gene inversion; grey shadow represents macrosyntenic patterns of genes in the same strand. The CSB-II sequence for the typical vertebrate mtDNA is a representation of the generally expected composition of the motif. The CSB-II sequences for the marine hatchetfish are the ones here identified. Unresolved regions with repeats (resulting for short-read assemblies) are represented in dashes.

The mtDNA lengths varied from 15 230 bp in *M. muelleri* to 23 291 bp in *A. aculeatus*, largely influenced by poorly resolved repetitive regions, which includes the putative CR and long species-specific intergenic regions (figures 1–3). The only previously assembled hatchetfish mtDNA with a CR annotation is *Polyipnus polli* Schultz, 1961 (AP012962.1) (figure 1). In other publicly available mtDNAs, the CR was either not annotated, i.e. in *Sternoptyx obscura* Garman, 1899 (OP057081.1) and in *Sternoptyx diaphana* Hermann, 1781 (MT588184.1) (figure 2), or not sequenced, i.e. in *M. muelleri* (AP012963.1) and *Argyropelecus affinis* Garman, 1899 (AP012964.1) (figure 1). The difficulty in obtaining this region has already been observed in many other animal groups (e.g. [26]). Conversely, in the three novel *Argyropelecus* sp*.* sequenced mtDNAs, i.e. *A. aculeatus*, *A. hemigymnus* and *A. olfersii*, long intergenic repeats were identified (figure 3). The difficulty in resolving these repetitive regions using short reads prevented the assembly of a single contig in *A. olfersii* (composed of two contigs), as well as the circularization of the *A. hemigymnus* assembly. This factor also hampered the assembly of the recently published *S. diaphana* mtDNA, which required scaffolding with Sanger sequencing [16]. Nevertheless, we efficiently identified the CSB-II (thus the CR) in all but one (i.e. *A. olfersii*) (electronic supplementary material, file S1 and figure S1).

Sequencing approaches are key for resolving complex mtDNA assemblies (e.g. [7,27]). Consequently, in *A. aculeatus* we used Oxford Nanopore long-reads to determine the composition of the complete mtDNA molecule. The produced reads were highly efficient in resolving the full mtDNA sequence, with some reads even spawning the entire molecule, supporting the inferred architecture. The final assembly was circularized, revealing two long repetitive regions, one spawning approximately 4800 bp, which included the CSB-II (between tRNA-Asp and tRNA-Pro) and the other approximately 1750 bp (between tRNA-Phe and a cox1) (figure 3; electronic supplementary material, figure S2). As for the other two *Argyropelecus* sp*.*, although the short-read assemblies retrieved all the mtDNA genes, the aforementioned repetitive regions were fragmented (figure 3).

### 2.2. Gene shuffling, translocations, duplications and inversions define the structure of mtDNA structure in deep-sea hatchetfish

We next investigated the gene content and overall structure of the mtDNA in deep-sea hatchetfish. The newly sequenced mtDNA as well as those deposited in GenBank show striking deviations from the standard vertebrate gene arrangement (figures 1–3). While the mtDNA from three species, i.e. *M. muelleri, A. affinis* and *P. polli*, maintain the standard vertebrate mtDNA architecture (figure 1), most of the newly sequenced mtDNA revealed considerably modified architectures (figures 3) [11,28]. The results show that shuffling, translocation and inversion of PCGs, rRNAs and more abundantly tRNAs, are widespread in the mtDNA deep-sea hatchetfish (figures 2 and 3). Even within the same genus, high structural differences can be detected (figures 2 and 3). Species from the *Argyropelecus* genus show four distinct mtDNA architectures: one maintains the standard vertebrate structure; while the other three share a radical inversion of a large fragment composed of several genes, with reciprocal additional unique features (figure 3). This fragment includes the inversion of the PCGs nad2 and nad1, the rRNA 16S (rrnL) and 12S (rrnS) and several tRNAs (M, I, L1, V and F), as well as the shuffling of two tRNAs (A and C) (figure 3). The inversion of two PCGs has never been reported in fish mtDNA before. To date, the only reported inversion of a PCG is of nad1 in a single clade of Antarctic notothenioid fishes (Nototheniidae: Trematominae) [17,29]. Interestingly, this also resulted from the inversion of a large fragment, which included the rRNA genes, several tRNAs (E, I, L2, V and F) as well as the CR [17,29].

Some species-specific tRNA duplications were observed (figures 2 and 3). The most striking of these duplications was captured by the Nanopore-based assembly in *A. aculeatus*, corresponding to a tandem duplication of two tRNAs C-A, followed by one tRNA C. This pattern seems to also occur in *A. olfersii*, in which a shorter tandem duplication of two tRNAs C-A was present, while in *A. hemigymnus* the two tRNAs are present in the same location but in a single copy (figure 3). Furthermore, *A. hemigymnus* shows a unique shuffling of the nad6 and the tRNA E (figure 3). The two *Sternoptyx* sp*.* revealed the overall same mtDNA architecture, with *S. obscura* having duplications and *pseudogenization* of nad3 and rrnL (figure 2). Gene duplication in mtDNA is often followed by the loss of one the copies, frequently retaining fragments of the original duplicated gene [11,17].

### 2.3. Phylogenetic analysis shows poorly resolved deep-sea hatchetfish evolutionary relationships

**Figure 4.**
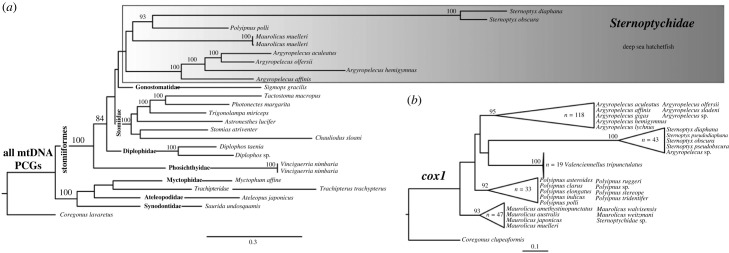
(*a*) IQ-tree maximum likelihood (ML) phylogenetic inference retrieved from the nucleotide alignment of the 13 mitogenomes concatenated protein-coding genes. (*b*) IQ-tree maximum likelihood (ML) phylogenetic inference retrieved from the nucleotide alignment of all available cox1 sequences of deep-sea hatchetfish in NCBI (electronic supplementary material, table S1). Above the nodes are represented ultrafast bootstrap values above 80%.

We next analysed the phylogenetic relationships between the various deep-sea hatchetfish species. The resulting phylogenetic inference, rooted with *Coregonus lavaretus* (Linnaeus, 1758) (following [16,30]), is split into two major groups, one composed of order Stomiiformes and the other composed of families Synodontidae, Ateleopodidae, Myctophidae and Trachipteridae (figure 4). Stomiiformes' phylogenetic relationships are poorly resolved, with very low support in most of the nodes. Within deep-sea hatchetfish, i.e. family Sternoptychidae, the relationships among the genus are also poorly resolved and an unexpected long branch is seen for the two S*ternoptyx* species (figure 4). The low support persisted in the amino acid-based phylogeny (electronic supplementary material, figure S3). The mtDNA phylogeny here presented is the first to include more than one species of the deep-sea hatchetfish (see [16]). However, the taxon representation is still incomplete, which likely explains the low support observed. The relationships of the four *Argyropelecus* sp*.* are well resolved and reflect the structural mtDNA variation here described (figures 1–3). *Argyropelecus affinis*, the only species to preserve the standard vertebrate structure is the first split within the genus, followed by *A. hemigymnus*, sister to *A. olfersii* + *A. aculeatus*, the two most structurally similar (figure 4).

To obtain a more taxa-representative phylogeny, all the cytochrome oxidase subunit I (COI) sequences available on GenBank were used (figure 4). The cox1 alignment included a total of 261 sequences (total length of 652 bp) (electronic supplementary material, file S2), from 26 distinct deep-sea hatchetfish species and the outgroup species, i.e. *Coregonus clupeaformis* (Mitchill, 1818). The resulting nucleotide phylogeny shows once again low support among inter-genus relationships (figure 4). Even though this phylogeny includes 260 sequences from marine hatchetfish, it highlights the need to increase the molecular data for the whole group.

### 2.4. GC/AT skew of strand-specific fourfold redundant sites show a strikingly frequent strand asymmetry reversal in deep-sea hatchetfish always coupled with CR strand relative position

The mtDNA asymmetric strand nucleotide composition of the H and L strands is generally highly demarcated in vertebrates, with a strong signature in the A-T and C-G composition of each stand [28]. This pronounced signature is the distinguishable factor between the two strands, i.e. the H(heavy)-strand, which is guanine rich, and L(light)-strand, which is guanine poor [31]. Conversely, inverted genes (i.e. genes that change to the complementary strand) will be exposed to the mutational bias specific of the new strand, and thus are expected to change their mutational signature accordingly, as proposed by the asymmetric model of mtDNA replication [12,15,32].

Given the newly detected inversions of two PCGs in *A. aculeatus*, *A. hemigymnus* and *A. olfersii*, as well as the oddly long branch lengths observed in *Sternoptyx sp.*, we next estimated the GC/AT skew of the PCGs at the fourfold redundant sites (the most likely to accumulate strand-specific mutations and to reflect the underlying mutational processes given that selection acting on these sites is weaker) for all the marine hatchetfish under study.

**Figure 5.**
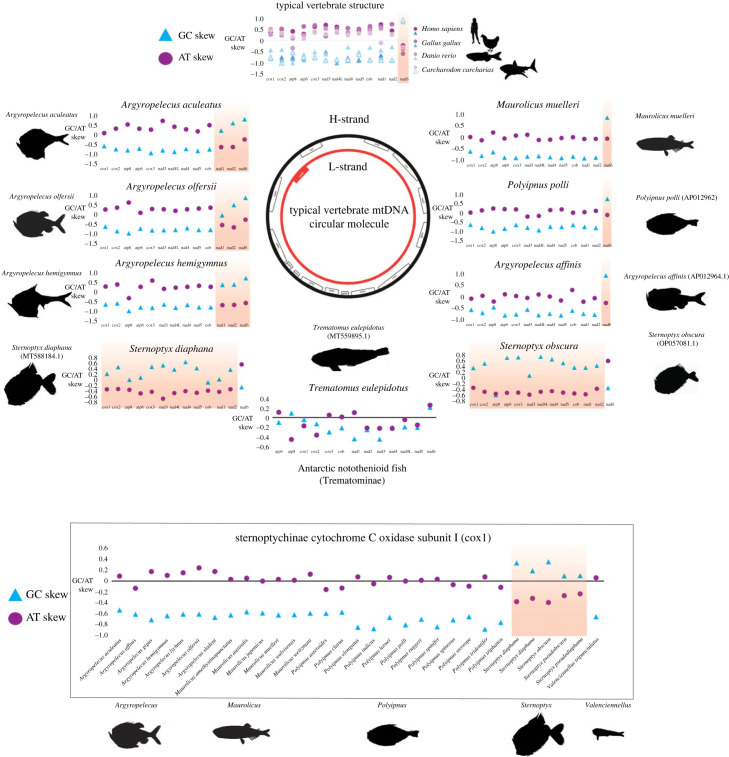
Top: strand-specific fourfold redundant sites GC/AT skew estimations for all protein-coding genes, for four model vertebrate species with standard vertebrate mitogenome gene order, each species of deep-sea hatchetfish analysed and one representative species from the Antarctic fish subfamily Trematominae that have an inversion of the ND1 and CR (inside the box). Bottom: strand-specific fourfold redundant sites GC/AT skew estimations for cox1 mitochondrial gene of all deep-sea hatchetfish, available in NCBI. Red shadows highlight the inversion of the strand-specific nucleotide GC/AT skew pattern. Middle: schematic of circular mitochondrial molecule highlighting the protein-coding genes encoded in the different strands. Positive numbers indicate an overabundance of Gs (blue triangles) or As (purple circles), while negative numbers indicate an overabundance of Cs or Ts.

**Figure 6.**
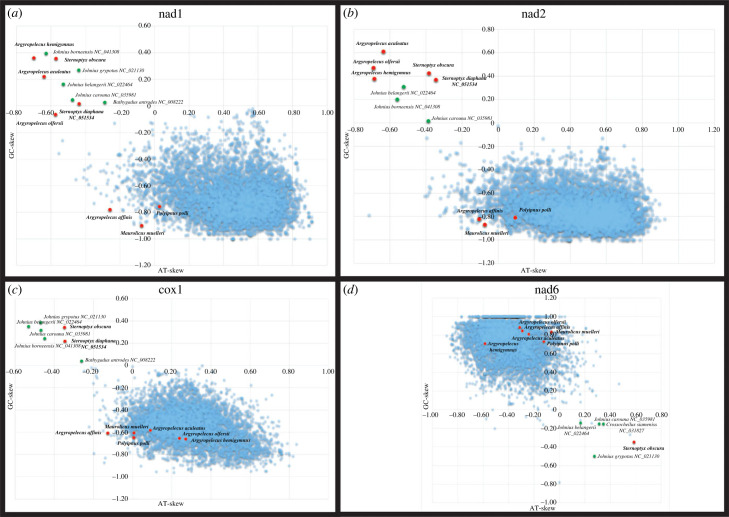
Plots of the GC and AT skews at the fourfold redundant sites of the mitochondrial protein-coding genes nad1, nad2, cox1 and nad6 from 6297 vertebrate species. Dots represent each of the 6297 analysed vertebrate species, with red (deep-sea hatchetfish) and green (other fish species) dots highlighting genes with inverted AT and GC skews. The plots for the remaining protein-coding genes are provided in electronic supplementary material, figure S4.

As expected, in the mtDNAs that maintains the standard vertebrate architecture with no detectable structural changes, i.e. *M. muelleri*, *A. affinis* and *P. polli*, shows the AT/GC skew pattern of a gene encoded in the L-strand (figures 5 and 6; electronic supplementary material, figure S4, i.e. AT skew < 0 and GC skew > 0). Interestingly, in the two *Argyropelecus* sp*.* with inverted PCGs polarity, this AT/GC skew pattern was observed in nad6, but also in nad1 and nad2, the two PCGs included in the inverted fragment (figures 5 and 6; electronic supplementary material, figure S4). Most strikingly, for the two *Sternoptyx* sp*.*, the entire AT/GC skew pattern is reversed in all PCGs (figures 5 and 6; electronic supplementary material, figure S4) but with no rearrangements or inversions detected in those genes. What could be causing the AT/GC skew reversion in these genomes? To date, such phenomenon, in vertebrates, has been suggested to result from CR inversion, and from the replication mechanism itself with the leading strand becoming the lagging strand and vice-versa during an asymmetric mode of replication [12,15]. We tested this hypothesis by characterizing the relative positioning of the initiation of replication and by identifying elements that have been described to be key in the replication mechanism of vertebrate mtDNA: the conserved sequence blocks CSB-II and III. Typically, CSB-II consists of six or more cytosines, one thymine and one adenine ending with five or more cytosines: 5′- CCCCCCTACCCCC-3′. We used this sequence as a reference, allowing for some variation in the length of the poly-C flanks. CSB-III is less conserved, generally identified by its positioning regarding CSB-II, and may be absent in fish CR [11]. The Motif Discovery analysis identified CSB-II in all the analysed sequences, except in *P. polli* while CSB-III was less prevalent, as previously observed by Satoh *et al*. [11] (electronic supplementary material, file S1 and figure S1). The absence of both CSBs in *P. polli* needs further evaluation, as this mitogenome is a direct submission to NCBI without detailed information on how it was generated. As previously hypostatized, the orientation of the CSB-II and therefore of the CR relative to the PCGs was the common denominator determining the AT/GC skew nucleotide asymmetry pattern (electronic supplementary material, file S1 and figures 1–3; electronic supplementary material, figure S1). That is, in the two *Sternoptyx sp.* the CSB-II is the only element changing its coding polarity (inversion), having the same polarity as nad6 and contrary to all other PCGs, while in the remaining species, the relative position follows the standard vertebrate architecture (electronic supplementary material, file S1 and figures 1–3; electronic supplementary material, figure S1).

To date, the only reported PCG inversion in a vertebrate is in one clade of Antarctic notothenioid fishes (Nototheniidae: Trematominae), consisting of the inversion of a large genomic segment (approx. 5 kbp), which includes four tRNAs, two rRNAs, the PCG inversion (nad1) and the CR [17,32]. Once again, the relative position of the CR, here identified through CSB-II (electronic supplementary material, file S1 and figure S1), is the determining factor shaping the nucleotide strand asymmetry, i.e. nad1 is the only PCG with the same polarity as the CR and thus it is also the only PCG maintaining its AT/GC skew pattern, while the remaining genes show a completely disrupted pattern. Interestingly, the fact that the latter-mentioned PCG shows a disruption but not a reversal AT/GC skew pattern, suggests that, in this group of organisms, the process of the mtDNA nucleotide composition strand asymmetry reversal is still in a transitory state (figure 5; electronic supplementary material, S5). A complete disruption of the typical strand asymmetry signature was detected in all 15 Trematominae mtDNA available, while the remaining notothenioid fishes maintained the standard pattern (electronic supplementary material, figure S5).

To access similar putative hidden patterns in other vertebrates, we next performed a vertebrate-wide assessment of the AT/GC skew at fourfold redundant sites [12,15] in a total of 6297 mtDNA (Retrieved from RefSeq in May 2022). The results demonstrate that the complete reversal of the nucleotide strand asymmetry composition is observed solely in the *Sternoptyx* sp*.* and those previously reported by Fonseca *et al*. [12,15], totaling seven species (figure 6; electronic supplementary material, figure S4). Moreover, single gene asymmetry reversal is detectable for the nad1 and nad2 genes of the three *Argyropelecus* species here described (figure 6; electronic supplementary material, figure S4). Given that there are still many un-sequenced marine hatchetfish whole mtDNA, we hypothesized that calculating the AT/GC skew pattern at fourfold redundant sites for the most sequenced mitochondrial gene of the group, i.e. COI, would be an informative strategy regarding the whole family. The results also show the reversed pattern in all analysed *Sternoptyx* species (*n* = 4), thus suggesting the reversal of the nucleotide strand asymmetry is shared by all analysed *Sternoptyx* (figures 5 and 6; electronic supplementary material, figure S4). The remaining hatchetfish species maintain the standard vertebrate AT/GC skew pattern, which is also in accordance with the results deduced from whole mtDNA assembly and annotation.

### 2.5. Genomic *oddities*: inverted mtDNA and large nuclear genomes

The combination of sequencing methodologies allowed the *de novo* assembly of mtDNA sequences from four species deep-sea hatchetfish species. Strikingly, the sequential analysis of gene annotation, phylogenetics, and AT/GC skew sequence investigations support a unique case of mtDNA structural plasticity [1]. We expanded the analysis to include thousands of available mtDNA. Our findings show that partial or full mtDNA inversions are evolutionary restricted to just two unrelated fish clades: *Argyropelecus* sp. and *Sternoptyx* sp. The immediate causes of this phylogenetically restricted alteration of mtDNA structure are unknown and deserve further exploration. In fact, within Metazoa, vertebrates show the strongest signs of purifying selection for gene rearrangement [1], with recent studies suggesting that gene blocks inversions (only recorded in invertebrates) promote sticking changes in the transcriptional pattern, thus requiring new regulatory elements [1,33–35]. Although the patterns here observed are likely to be the result of a relaxation of selection, they also raise the exciting possibility of an adaptive scenario of the reported mtDNA oddities. Interestingly, using the high-coverage whole genome sequencing for *A. aculeatus* and *S. diaphana* we were able to appraise the genome size for both species (electronic supplementary material, figure S6). The results show an unexpectedly large genome size estimation, with both species being approximately 2.70 Gbp long (electronic supplementary material, figure S6). Most teleost species have an average of 1 Gbp long genome size, with the exceptions being usually attributed to whole genome duplication events [36]. Together, our results suggest that like the mitogenomes, the whole genome of deep-sea hatchetfish is also unusual and deserves future explorations.

## 3. Methods

### 3.1. Samples collection and DNA extraction

In total, four specimens were captured for this study, three from the genus *Argyropelecus*, i.e. *Argyropelecus aculeatus* Valenciennes, 1850, *Argyropelecus hemigymnus* Cocco, 1829 and *Argyropelecus olfersii* (Cuvier, 1829), and one from genus *Maurolicus*, i.e. *Maurolicus muelleri* (Gmelin, 1789). The specimens were obtained during the scientific surveys: EU Groundfish Survey (Platuxa-2019) in North Atlantic (43.1544 N–51.4429 W, 2019); from the EU Groundfish Survey (Platuxa-2019) in the Northwest Atlantic Ocean (43.3838 N–49.0036 W, 2019); from the Survey PORCUPINE20 in Porcu-pine Bank (51.0677 N–14.2862 W, 2020) and from EU Groundfish Survey FN3L19 in North Atlantic (47.4978 N–47.6680 W), respectively. Morphological identification was performed onboard and whole specimens were stored in absolute ethanol and are stored at DNA and Tissue bank at CIIMAR—Interdisciplinary Center of Marine and Environmental Research. The specimen treatment has been approved by the CIIMAR ethical committee and by CIIMAR Managing Animal Welfare Body (ORBEA) according to the European Union Directive 2010/63/EU. Whole genomic DNA for each specimen was obtained from a small portion of the muscle tissue using the Qiagen MagAttract HMW DNA extraction kit, following the manufacturer's instructions. For all samples, total DNA was used for Illumina paired-end (PE) library preparation and sequencing at the Macrogen, Inc. (Seoul, Korea), using Illumina HiSeq X Ten platform, with 150 bp PE configuration. For *A. aculeatus* and *S. diaphana* high coverage whole genome (WGS) PE sequencing was performed, while the remaining samples were only sequenced at low coverage. Furthermore, low-coverage Nanopore (Oxford Nanopore) genome skimming was performed for *A. aculeatus*. Briefly, approximately 1 µg of genomic DNA was used for library preparation using the ligation library prep kit LSK109 and after sequenced on an FLO-MIN106 revD SpotON R9.4 Flow Cell for 48 h.

### 3.2. Whole mitogenome assemblies and annotation

Raw Illumina PE reads were quality-filter and adaptors were removed using Trimmomatic (version 0.38) [37], using the parameters LEADING:5 TRAILING:5 SLIDINGWINDOW:5:20 MINLEN:36. Read quality was inspected before and after trimming using FastQC (version 0.11.8) (http://www.bioinformatics.babraham.ac.uk/projects/fastqc/).

Mitogenome reconstruction for each species was obtained using several distinct approaches. For each dataset, several preliminary assemblies were performed using distinct approaches, including whole genome assembly (e.g. using SPAdes, [38], MetaSPAdes [39] and MEGAHIT [40]) followed by mtDNA fishing, as well as reference baiting assembly approaches (e.g. using NOVOPlasty [41], MITObim) [42] and GetOrganelle [43]), using single genes and whole mtDNA. Given that no approach revealed to be transversely efficient for all datasets, bellow we described the most efficient workflow, which assures confidence in the resulting assemblies, and thus may be replicated in future attempts to assemble similar mtDNA using Illumina short reads. For each species, assemblies were first performed with GetOrganelle v1.7.1 [43], specifying a multi k-mer approach (i.e. from 20 to 125 with a 5-mer increment). GetOrganelle is an interactive baiting pipeline that filters mtDNA reads and uses the SPAdes assembler [38] to reconstruct the mitogenome with the filtered reads. The results of the assemblies were individually validated using multiple approaches. For cases where multiple assemblies were generated, i.e. *A. hemigymnus*, and *A. olfersii*, the gfa files were inspected using Bandage v0.8.1 [44] which revealed several ambiguous disjoints. Furthermore, annotation was generated for some of the putative assemblies using the module ‘annotate’ from MitoZ v.3.4 [45], which showed that the ambiguous disjoints were localized within no-coding repetitive regions. Consequently, new assemblies were performed, first using larger k-mer with GetOrganelle v1.7.1 and if the problem persisted, using metaSPAdes v3.12.0 [39] with the maximum K-mer size possible (i.e. 127). In the end, the selected best representative assemblies for each species were as follows, for *M. muelleri* the GetOrganelle assembly with multi k-mer (20–125-mer with a 5-mer increment), for *A. olfersii* the GetOrganelle assembly single k-mer (131-mer) and for *A. hemigymnus* the metaSPAdes assembly with the maximum allowed k-mer size (127-mer). Every generated assembly was annotated with MitoZ (as described above). Read coverage distributions were analysed by aligning PE reads to the final genome assemblies using the Burrows–Wheeler Aligner (BWA) v.0.7.17-r1198 [46] and visualized in Artemis v17.0.1 [47] (electronic supplementary material, figure S7).

The Nanopore reads of *A. aculeatus* were quality-filtered using Filtlong (https://github.com/rrwick/Filtlong). Given that long repetitive regions seem to cause problems with the PE-based assemblies, the Nanopore reads were filtered by size (i.e. >21 000 bp) to include only reads spanning the whole mitogenome. The mitogenome assembly was performed using Unicycler v.0.4.8. [48]. The assembly was polished, following the author's suggestions, with the Nanopore reads using medaka v1.2.2 (https://github.com/nanoporetech/medaka) and with short-reads, first using Polypolish v0.4.3 [49] and after using Polca [50]. Genome annotation was performed with MitoZ (as described above). Read coverage distributions were analysed by aligning PE reads (as described above) and with Nanopore reads using Minimap2 v.2.24 [51].

Finally, since the *Sternoptyx obscura* mitogenome available on NCBI (OP057081) is marked as ‘UNVERIFIED’ and therefore no annotation is provided, the mitogenome was downloaded and annotated using MitoZ (as described above).

To identify the putative CR of the deep sea hatchetfish mitogenome assemblies, we search for conserved motifs within non-coding regions to identify any of the known CSB that are involved in the replication initiation (Satoh *et al*. [11]). The recently described CSBs of several fish species were here used as a reference to guide the search, including one Stomifformes, i.e. *Diplophos taenia* (Satoh *et al*. [11]). We focused on the two most conserved of the three CSBs, i.e. CSB-II and CSB-III. Given the lack of significant non-coding regions within the mitogenomes of *M.muelleri*, *A. affinis* and *A. olfersii* (see 'Results and discussion')*,* these mitogenomes were not included in the analysis. Moreover, given the highly disproportionate read coverage distribution in the mitogenome non-coding regions of *A. hemigymnus* (electronic supplementary material, figure S7), this mitogenome was not considered. The coverage distribution indicates regions probably represented by the collapse of a highly repetitive region, and thus not suited for the analysis. The non-coding regions from the remaining deep-sea hatchetfish species, the Antarctic Trematominae species, *Pagothenia borchgrevinkias* (with the only other record CDS mitochondrial inversion), as well as all the fish CSB-II and CSB-III identified by Satoh *et al*. [11] were used. The sequences were uploaded to MEME v.5.5.2 [52] and the Motif Discovery model was used to identify conserved motifs across all sequences, specifying a maximum motif width of 25.

### 3.3. Phylogenetic reconstruction

To produce a phylogenetic reconstruction, the mitogenomes of all Stomiiformes available on NCBI (*n* = 15), including four Sternoptychidae species (i.e. *S. obscura*, *M. muelleri*, *P. polli*, *A. affinis*), the mitogenomes here produced and four outgroup taxa were used (table 1). Alignments of the 13 PCGs were produced using MAFFT v7.453 [53]. Positions with gaps in 50% or more of the individual alignments were removed using trimAL v. 1.2rev59 [54] and all alignments were concatenated using FASconCAT-G (https://github.com/PatrickKueck/FASconCAT-G). The alignment composed by the concatenated PCGs from 15 species had a total length of 11 436 bp. The partition scheme and the evolutionary models that best fit those schemes, as well as maximum likelihood (ML) phylogenetic inference were produced in IQ-TREE v.1.6.12 [55,56].

For the amino acid phylogenetic reconstruction, individual alignments were translated to proteins using the EMBOSS seqret V.6.6.0.0, trimmed with trimAL v. 1.2rev59 (as described above) and concatenated using FASconCAT-G. The alignment composed by the concatenated PCGs from 15 species had a total length of 3801 aa. The partition scheme and the evolutionary models that best fit those schemes, as well as ML phylogenetic inference were produced in IQ-TREE v.1.6.12.

### 3.4. COI phylogeny

To produce a phylogenetic reconstruction with a wider taxa representation, all cox1 sequences available on GenBank (*n* = 261) were downloaded (including the outgroup taxa *C. clupeaformis*) (electronic supplementary material, table S1). Alignment was performed using MAFFT v7.453 [53] and an ML phylogenetic inference was constructed in IQ-TREE v.1.6.12, also estimating the best evolutionary model for the analysis.

### 3.5. Strand-specific fourfold redundant sites GC/AT skew estimations

The PCGs of the complete mitogenomes of all vertebrates available in GenBank (http://www.ncbi.nlm.nih.gov) in May of 2022 were retrieved. The calculation of the GC skew (G-C)/(G + C) and the AT skew (A-T)/(A + T) at fourfold redundant sites using custom Perl scripts [15]. The redundant codons examined were alanine (GCN), proline (CCN), serine (TCN), threonine (ACN), arginine (CGN), glycine (GGN), leucine (CTN) and valine (GTN).

Furthermore, to infer GC/AT skew inversions in other species from family Sternoptychidae, for which no whole mitogenome is available, the COI sequences for all Sternoptychidae species available on GenBank were downloaded and the calculations applied, as described above.

### 3.6. Whole-genome size estimation

The high coverage WGS PE sequencing reads for *A. aculeatus* and *S. diaphana* were used to estimate the overall characteristics of each species' genomes using Jellyfish v.2.2.10 and GenomeScope2 [57] with a k-mers length of 21.

## Data Availability

The raw sequencing reads and mtDNA assemblies are deposited at NCBI, and respective SRA and assembly accessions are depicted in table 1, all linked to BioProject PRJNA977192. Mitogenome assemblies’ accessions are OR062951–OR062955. Supplementary material is available online [58].
